# Evaluation of Ginkgo Biloba Extract on Hematological Changes Affected with Hazards of Electromagnetic Field Exposure

**Published:** 2009-09

**Authors:** H. E. Abdel Baieth

**Affiliations:** *Department of Physics, Faculty of Science, King Faisal University, Saudi Abrabia Kingdom*

**Keywords:** ginkgo biloba, free radicals, EMF-viscosity of blood, dielectric properties of hemoglobin

## Abstract

The aim of the present work is to study if the GBE 761 (Ginkgo biloba leaves extract) which is beneficial in arterial disease owing to its vasodilator and blood flow acts against the hazards of exposure to electromagnetic field. Here, the GBE was used in two ways either as a protector or for treating the hazards due to exposure to electromagnetic field (EMF). For this purpose, albino rats were grouped into six groups and blood samples were collected from eye vein of the animals from all groups at the end of the experiment. This study concentrates on the cellular membrane and function of the RBCs and focuses on the rheological and physical measurements for blood and hemoglobin molecule because the RBCs membranes play an essential role in the blood flow rate. The changes in its biophysical properties of RBCs membrane will affect its capability for carrying on its metabolic functions. Furthermore, the molecular diameter of hemoglobin, its relaxation time and conductivity were calculated from the dielectric relaxation data. The results indicate that the administration of GBE led to the decrease of RBCs membrane elasticity will lead to the increase of the blood viscosity. Results suggest that GBE may be not of clinical value as anti-oxidant drug for such diseases occurred due to to EMF exposure for long time. It may be conclude that through treatment with those extract some physical measurements like “viscosity of blood, Osmotic fragility to measure the hemolysis rat of RBCs, the radius and the conductivity of hemoglobin molecule” should be considered during the time of treatment. Overall, these leaves (GBE) need more study and there is a recommendation to put the physical parameters parallel to the clinical study.

## INTRODUCTION

Over the past few years, considerable attention has been given to the potential bio-effects of Electromagnetic field (EMF) due to increasing use of electric power for domestic and industrial appliances. One important fact, the electromagnetic field (EMF) interacts with living systems and many studies have suggested that EMF may increase the risk of various types of blood diseases including leukemia, brain and brest tumours (18, 21, 23, 27).

The most recognized hypotheses for the biophysical mechanism of EMF is the radical pairs mechanism which formed free radical species (oxygen-free radicals) in the cells. These free radicals have been considered the mediators of the excessive lipid peroxidation decline of membrane fluidity, and cell damage observed in Al-Zheimer's disease (15).

The Ginkgo biloba leaves extract (GBE 761) which is a standardized extract from Ginkgo biloba leaf (15), used related to its antioxidant properties and act as free radical scavenging (14), rheological (17, 19). Also the GBE used for its beneficial effects on brain functions, particularly its memory enhancing properties and is being used in the diseases such as Al Zheimer or in people experiencing age-related decline in memory (10, 20) due to vascular or degenerative dementias.

While the characteristic biological effects of EMF appear to be functional changes in the central nervous system (5), ginkgo biloba is described as neuroprotective (2, 8) and beneficial in arterial disease, perhaps owing to its vasodilator (22) and blood flow (6).

Pharmacological effects of the extracted related to its free radical scavenging properties include inhibition of lipid peroxidation, helping to maintain integrity and permeability of cell walls (16) and protection of brain neurons against oxidative stress and post-ischemic injury induced by free radicals due to the exposure to electromagnetic field.

Were the free radicals which induced from chemicals different from those due to EMF exposure?

Do the compounds in the Ginkgo act pharmacologically as scavengers for free radicals resulted from the exposure to EMF?

## MATERIALS AND METHODS

### Animals

In the present investigation forty-two male Wistar albino rats weighing 120–130 g of body weight used. These were divided into sex groups: the normal group which is neither exposed to EMF nor treated with the GBE extract, group 1 was used for direct effect studies (animals of this group were exposed continuously to magnetic field of 0.3 mT (50 Hz) for 21 days, this group was exposed without receiving any treatment). Group 2 exposed to EMF three weeks continuously and received GbE for one week after exposure. Group 3 received GbE for one week before exposure to EMF for three weeks. Group 4 exposed to EMF and received GbE together for three weeks (by the same dosage of control groups). Group 5 was control (received the Ginkgo-biloba (GbE)) for three weeks without exposure to electromagnetic field and finally the normal group which housed in a similar cage, kept during the run of the experiment in a typical cylindrical chamber. The temperature inside the laboratory varied between 25 and 27°C. Lighting conditions were natural light from large windows during the day and complete darkness during the night. In order to mitigate the problem of contamination of the magnet, the magnet was washed, rinsed, dried and furnished with clean dry, large grain saw-dust, and every 24 hours. Food was available daily or (24 h/day) and the animals received the same diet during the course of the experiment. At the End of exposure period, blood samples were directly collected from the eye-vein of the rat using a heparinated capillary tube. The heparinized blood was centrifuged to 3500 r.p.m. for 10 minutes at 4°C, then the plasma was taken out and stored at 20°C to give hemoglobin (Hb) following the method cited by Trivelli *et al* 1971 (26). There are no restrictions in Egypt for the use of experimental animals for laboratory studies.

### Exposure facility

Animals exposed to a homogenous magnetic field generator manufactured locally in Cairo University Workshop (3). The magnetic field intensity was measured by means of a hand held Gauss/Tesla meter (model 4048 with prop T-4048 manufactured by (W. Bell) in U.S.A of accuracy 0.2%.

### Dosage and Routine of Administration

GbE solution (25 mg/Kg^−1^) was prepared by dissolving 2.25 mg in 10 ml distilled water and was given to animals orally (1 ml/100 mg) body weight (1) once a day for one week after exposure (group 2), for one week before exposure (group 3), for three weeks during exposure (group 4) and for three weeks without exposure (group 5).

**Osmotic Fragility.** Osmotic fragility curves of whole blood have been plotted for control and experimental groups according to *Dacie and Lewi* (7). The test was carried out within two hours of collection of blood. Whole blood is added to varying concentrations of buffered sodium chloride solution NaCl buffered to pH 7.4 and kept at 25°C. The amount of hemolysis is then determined by reading the supernatants using a spectrophotometer (UV/visible spectrophotometer Jasco V-530, made in Japan) (4).

**Viscosity Measurements.** In order to determine the rheological properties of blood, it is necessary to use an instrument in which all the samples under test is exposed to a uniform shearing stress and shearing rate, and each of these effects is separately determinable. The rheometer used in this study is the Brookfield DV-III Programmable Rheometer.

The Brookfield DV-III Programmable Rheometer (cone and plate) measures blood parameters of shear stress and viscosity under controlled conditions of shear rates, temperature and time. The sample volume required for this experiment was 0.5 ml. of fresh heparinized blood. Computer controlled measurement protocols allow for ease of operation and reproducible measurement conditions. According to the principle of the cone-plate rheometer, rotation of a flat cone upon a plane surface (the plate) at different speeds of rotation (different shear rate) takes place. The shear rate of a given measurement is determined by the rotational speed of the spindle, the size and shape of the spindle, the size and shape of the container used, and therefore, the distance between the container wall and the spindle surface. The data was collected from the rheometer by means of software program.

**Hemoglobin spectra**. Hemoglobin spectrum was made by using the spectrophotometer (UV/visible spectrophotometer Jasco V-530, made in Japan). It was made in the range 300 nm to 700 nm at room temperature (25°C).

**Dielectric properties.** Hemoglobin (Hb) solution was prepared according to the method of (9). Dielectric measurements were made on the collected hemoglobin from the different groups of rats. Dielectric measurements were made in the frequency range from 50 Hz to 200 kHz using LCZ meter type Chen Hwa 1061 manufactured by Taiwan IEEE-488 interface, with a sample cell of platinum black electrodes of dimensions (0.2 cm × 0.3 cm). During measurements, the samples within the cell were kept in a temperature-controlled system at 25 ± 0.1°C. The measured values of capacitance (c) and resistance (r) were used to calculate the real (ε′) and imaginary (ε″) parts of the complex permittivity and the conductivity σ.

The dielectric constant (ε′) and ac conductivity (σ) for the samples were calculated at each frequency from the measured value of the capacitance (c) through the equation:

(a) $${ɛ}^{\prime }=c/K{ɛ}_{o}$$

where *K* is the cell constant which is a function of cell dimensions, *ε_o_* is the permittivity of free space (11).

The dielectric loss ε″ was calculated from the relation:

(b) $${ɛ}^{\prime \prime }={ɛ}^{\prime }\;\text{tan}\delta \;\text{and}\;\text{tan}\;\delta =1/2\pi \text{fRC}$$

The conductivity *σ* is calculated by the equation:

(c) $$\sigma =K/R\;(\Omega^{-1}m^{-1})$$

where, *R* is the measured resistance of the samples. This implies that the permittivity and conductivity are not independent with frequency. The conductivity σ has usually a frequency independent part (due to ionic conduction) and a frequency dependent part (due to dielectric relaxation). The relaxation time (τ) can be calculated from the relation:

(d) $$\tau =1/2\pi f_{c}$$

where *f_c_* is the critical frequency corresponding to the mid point of the dispersion curves. The radius (r) of the molecule is given by:

(e) $${\text{r}}^{3}=\text{KT}\tau /4\pi \eta$$

η is the viscosity of the sample. The dielectric relation in the frequency range of *kilo* hertz and below was known by the ∝-dispersion (12), is mainly due to counter ions molecules of hemoglobin.

The relaxation time is proportional to the square of the molecule radius:

(f) $$\tau ={\text{er}}^{2}/2\mu \text{kT}$$

where, µ is the surface mobility of the counter ions (m^2^/V sec), e is the charge of the counter ion, k is Boltzman constant and T is the temperature of the sample in Kelvin. When an electric field is applied, the ions in the system will redistribute under the influence of both, the field and the diffusion of ions (12, 13). The time constant τ of this dispersion is similar to that derived by *Schwarz* (24).

(g) $$\tau ={\text{r}}^{2}/\text{D}$$

where *D* is the diffusion coefficient of ions.

**Statistical analysis.** All results have been done for animals of all groups; the average readings of each group were used to calculate the means and standard deviations according to *Anova*. After calculation of the *t* value and consultation of the *t* distribution table, probability value p was calculated. P>0.05 was considered not significant (NS), for 0.05>P>0.01, the difference is significant (s), and for 0.01≥p>0.001, highly significant, and for 0.001≥p, very highly significant (HS).

## RESULTS AND DISCUSSION

### Influence of EMF and GBE on the optical spectra of hemoglobin

Table 1 illustrates the hematological data from rats for all groups: RBCs: Red blood cells. MCV: Mean Corpuscular volume. MCH: Mean Corpuscular Hemoglobin. MCHC: Mean corpusclar Hemoglobin concentration. HCT%: Hematocrite percentage. Hb: Hemoglobin. TLC: The white blood cells. The results indicate that there is a significant decrease were observed as regards in count total red blood cells to about 50% in the group 5 which received only the GbE solution and not exposed to EMF and this associated with the reduce of Hb, while the mean corpuscular volume MCV increased to about 100% in the same group and this is naturally occurring as a step before hemolysis occurring (HCT%). There is also a remarkable increase in TLC in the groups 3 and group 5 which received the GbE solution even before exposure to EMF.

**Table 1:** Illustrate the hematological data from rats for all groups

	Control	Group 1	Group 2	Group 3	Group 4	Group 5

Hb (gm/dl)	12.2 ± 0.01	11.65 ± 0.06	11.5 ± 0.02	11.2 ± 0.02	14.8 ± 0.06	8.9 ± 0.04
RBC (melion/*ul*)	5.8 ± 0.012	4.7 ± 0.05	4.14 ± 0.002	4.0 ± 0.032	5.3 ± 0.05	3.2 ± 0.02
TLC (x100/*ul*)	9.2 ± 0.9	9.9 ± 0.9	8.13 ± 0.003	12.5 ± 0.06	9.4 ± 0.01	12.8 ± 0.02
HCT %	37.2 ± 1.1	41.2 ± 0.09	0.345 ± 0.005	0.37 ± 0.005	0.44 ± 0.006	0.37 ± 0.006
MCV (fl)	61.9 ± 0.01	87.04 ± 0.04	83.4 ± 0.01	92.51 ± 0.009	83.3 ± 0.03	117.3 ± 0.04
MCH (pg)	20.9 ± 0.02	29.01 ± 0.04	27.74 ± 0.03	27.7 ± 0.07	27.8 ± 0.05	27.8 ± 0.01
MCHc (gm/dl)	33.7 ± 0.01	33.3 ± 0.025	33.3 ± 0.003	30.17 ± 0.0007	33.3 ± 0.04	23.8 ± 0.02

**Figure 1:**
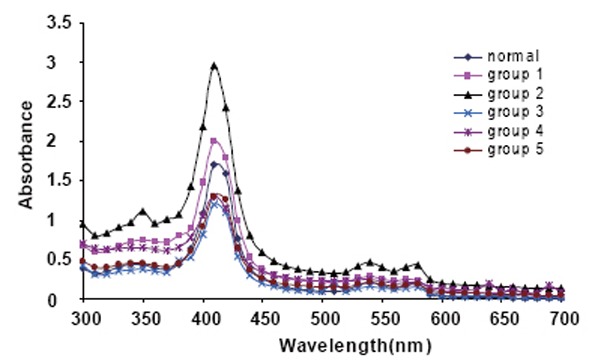
The absorption spectra of Hb molecules for all groups.

Figure 1 illustrate the spectra of Hb for all rats' blood samples in the range between 400–620 nm and show three absorption peaks at 410 nm, the soret band, characteristic peak of iron and two maxima from 500 to 600 nm, they are attributed to heme-heme interaction and spin state of iron.

The peak height and the absorption ratios in the absorption spectra for hemoglobin extracted from the collected blood from all groups are given in Table 2. The change in the intensities could represent changes in the number of molecules involved in these processes.

**Table 2:** Peak height (410) for Hb spectrum and absorption ratio (A_580_ / A_540_)

Group	*Peak height of (A_410_)*	*Absorbance ratio(A_580_ / A_540_)*

Normal	1.71	0.6423
Group 1: Exposed (3 mT), 21 days	1.99	0.70143
Group 2: Exposed first to EMF and then treated with GBE	2.95	0.91216
Group 3: Treated first with GBE and then exposed to EMF	1.24	0.9077
Group 4: Treated with GBE through exposure to EMF	1.34	0.8859
Group 5: Treated with the GBE only without exposure to EMF	1.48	0.8859

The ratio *A_580_ / A_540_* shows no linear relation with different groups and this indicate changes in hemoglobin interaction bond which seem to be weaker and the distribution of these molecules was brooder as compared with control. Since the absorption peaks *A_540_* and *A_580_* are both affected by the treatment with GBE and he increase in the ratio (*A_580_ / A_540_*) indicate that the transformation of hemoglobin molecule to the oxidized form (methemoglobin).

### Influence of EMF on red cell deformability

**Figure 2:**
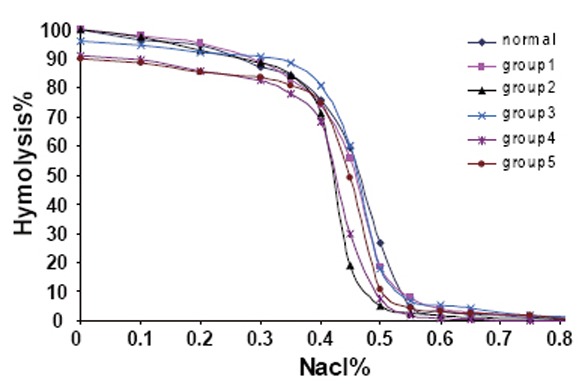
The variation of the prcentage of hemolysis for the RBCs as a function of the NaCl% for samples collected from animals from all groups

**Figure 3:**
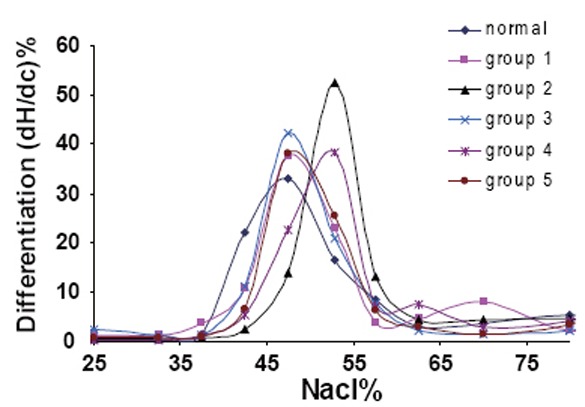
Differentiation curves for all groups

The effects of GBE as treatment or as protected from the hazards of exposure to electromagnetic field exposure will be seen in the measurements of the cell rigidity, permeability and elasticity. The red cells can be modified by hardening and by changing osmotic pressure (Figure 2), which show the effects of GBE and electromagnetic field on the osmotic fragility measurements of RBCs collected from animals of the different groups. By the addition of hypotonic saline to swell the cells, the percentage of hemolysed cells is plotted as a function of the concentration percentage of NaCl. For analysis of these results, the curves were differentiated and plotted as a function of NaCl concentration percentage as shown in Figure 3. As a result of this treatment each characteristic plot in Figure 2 was represented by a peak in Figure 3 whose width indicate the elastic range of the RBCs cellular membrane.

The percentage of NaCl concentration C% at which hemolysis starts to occur characterizes the transport of water molecule through the RBCs membrane and hence its permeability. The widths at half maximum W_hmax_ of these differential plots represent the relative elastic limit of RBCs membrane. The increase of W_hmax_ will represent the increase of cellular membrane elasticity. The average value of C% and W_hmax_ for RBCs from each group are given in Table 3.

**Table 3:** The average value of C% and W_h max_ for RBCs from each group are given (male rats)

Group	*C% permeability*	*W_h max_* Elasticity

Normal	35%	0.2
Group 1: Exposed (3 mT), 21 days	30%	0.25
Group 2: Exposed first to EMF and then treated with GBE	35%	0.15
Group 3: Treated first with GBE and then exposed to EMF	35%	0.2
Group 4: Treated with GBE through exposure to EMF	40%	0.1
Group 5: Treated with the GBE only without exposure to EMF	40%	0.1

The data indicates that the permeability of RBCs decreased only with the exposure to the EMF while at this dose the cellular membrane elasticity increases. There were also remarkable increases in the permeability of groups 4 & 5 which treated with the GBE and this increase of permeability associated with decrease in the elasticity in the same groups and this gives reasons for this study. While the exposure to EMF decreases the permeability, this did not happen in the case treated with the GBE in spite of that it gives a reversible result. This remarkable decrease in the elasticity of the RBCs membrane may lead to hemolysis of the RBCs which growing resistance of the blood capillaries to the passage of the RBCs and hence toxicity in some organs could occur. These results lead us to study the viscosity.

### Influence of EMF on Viscosity

**Figure 4:**
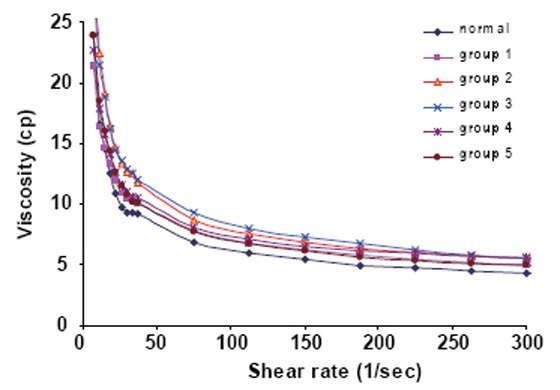
The variation of te blood viscosity as a function of shear rate for all groups.

Figure 4 illustrates the shear rate dependence of the blood viscosity in (cP) of normal blood and exposed samples before and after treating with the GBE extract (all groups).

Thurston 2004 (25) investigated that the hematocrit HCT, is strong parameter in establishing the viscosity of blood because of its central role of the elastic red cell and normally there is a proportional relation between HCT and viscosity.

Table 4 illustrates the hematological data for all groups to see how the viscosity changes with hematocrite in the presence of electromagnetic field.

**Table 4:** The average value of (permeability) HCT% and Viscosity (cp) for all groups

Group	*HCT% (permeability)*	Viscosity (cp)

Normal	0.36	0.0368
Group 1: Exposed (3 mT), 21 days	0.3918	0.0437
Group 2: Exposed first to EMF and then treated with GBE	0.3456	0.0473
Group 3: Treated first with GBE and then exposed to EMF	0.3705	0.0477
Group 4: Treated with GBE through exposure to EMF	0.44594	0.0525
Group 5: Treated with the GBE only without exposure to EMF	0.3768	0.0436

It was observed from that figure that the viscosity increased as the hematocrite HCT% increased in the group exposed first with the electromagnetic fields. There is a remarkable increase in the viscosity of group 4 which received the extract through exposure to EMF and also in group 5.

This may be explained as “The plasma proteins especially fibrinogen is the main determinants of the blood viscosity” which may reduce under the effect of electromagnetic field.

One may conclude that, the decrease of RBCs membrane elasticity will lead to the increase of the blood viscosity and this is confirmed from the results. We can also recommend with the following: the patients who treated with GBE shouldn't expose to EMF and should the diagnostician examine his blood viscosity in a period of time through the treatment time.

### Influence of EMF on the molecular structure of Hb

**Figure 5:**
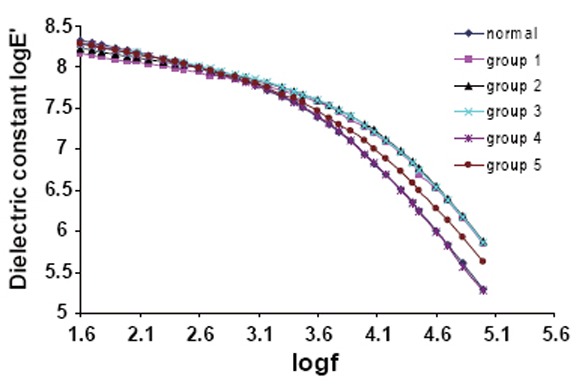
The variation of the dielectric constant E’ for Hb from all groups as a functional of applied frequency.

Figure 5 shows the variation of the dielectric constant (ε′) as a function of the applied frequency for Hb samples collected from all groups. It is clear that this dispersion arises from polarization of counter ions the membrane surface. The results indicate a strong dispersion in the β region for all the samples from all groups studied. The dielectric dispersion in the β range (0.1–5 MHz) is mainly due to protein and counter ion molecular relaxation.

**Figure 6:**
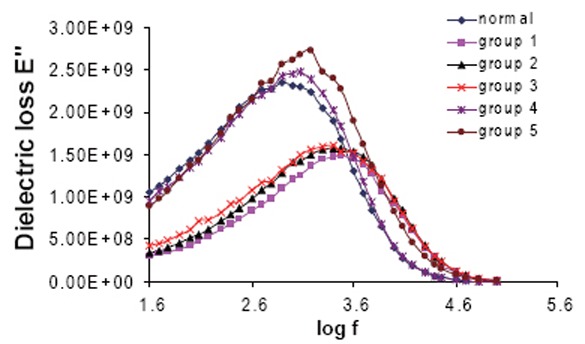
The variation of the dielectric loss E" of Hb for all groups as a function of frequency

**Figure 7:**
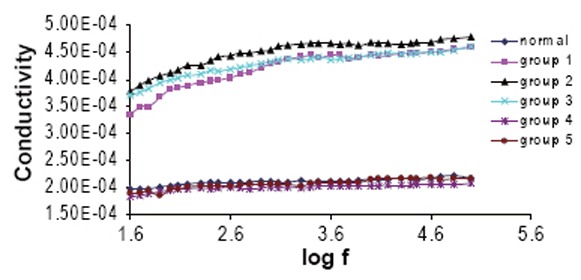
The variation of the conductivity of Hb for all groups as a function of frequency

Figure 6 and Figure 7 show the variation of the dielectric loss (ε″) and the conductivity (σ) as a function of frequency for all groups. The average values for relaxation time (τ), molecular radius r and conductivity σ were measured and given in Table 5.

**Table 5:** The average values of the relaxation time (τ), radius (r) and conductivity (σ)

Group	τ (μ SEC)	R(NM)	σ(S/M)

Normal	0.79 ± 0.02	6.88 ± 0.003	0.18 ± 0.006
Group 1: Exposed (3 mT), 21 days	1.59 ± 0.01	13.5 ± 0.015	0.231 ± 0.07
Group 2: Exposed first to EMF and then treated with GBE	0.531 ± 0.02	11.9 ± 0.015	0.212 ± 0.005
Group 3: Treated first with GBE and then exposed to EMF	0.717 ± 0.05	13.1 ± 0.05	0.215 ± 0.01
Group 4: Treated with GBE through exposure to EMF	1.19 ± 0.003	15.6 ± 0.003	0.11 ± 0.01
Group 5: Treated with the GBE only without exposure to EMF	1.46 ± 0.003	16.6 ± 0.003	0.103 ± 0.01

**The Effect of EMF exposure alone.** The result indicate that exposure of animals to EMF for three weeks (group 1) caused structural changes in Hb molecule, where both the relaxation and the radiuses of the Hb molecules increased to about 200% and the conductivity increased to about 128%.

**The Effect of GBE alone.** When the GBE was oral given at 25 mg kg^−1^ once a day for three weeks without any effect of EMF, there was a significant effect on the Hb molecule structure (group 5).

The relaxation time increased to about 184% while the radius of Hb molecule increased to about 240%. Furthermore, the conductivity reduced to about 57%.

While oral administration of GBE inhibited the carageenin induced paw oedema (1).

The elasticity of RBCs decreases to about 50% and the permeability increases which led to more hemolysis and then increase in blood viscosity to about 130% than normal.

**Administration of GBE after the exposure to EMF.** Beginning administration of GBE at the end of exposure to EMF period (group 2) give the following results, the relaxation time decrease to about 67% and this was a reason why the conductivity increased. Furthermore, there was no change in the radius of the Hb molecule computable to the normal because of the increase of the permeability that causes more diffusion of the fluids through the cell membrane.

**Administration of GBE before the exposure to EMF.** Administration of GBE with dose 25 mg kg^−1^ once a day for one week before exposure to EMF (group 3) may be use as a protector or antioxidant. The results indicate that there was increase in the viscosity associated with increase in hemolysis.

In addition, the radius of Hb molecule increased as nothing affect (group 1) and the conductivity increased because of EMF.

**Administration of GBE through exposure to EMF.** It noted that the relaxation time of this group 4 decreased compared to that of both group 1 and group 5 where the radius of the Hb molecule increases.

## CONCLUSION

It may concluded that the Hb molecule tend to resist what happened through the exposure to the magnetic field which resulted in the decrease of the membrane elasticity of RBCs which play a role in the blood viscosity. It may conclude also that it is important to know the reasons of some diseases, if such dieses resulted from EMF exposure as “Al Zaheimer” it should not treated with the GBE. Overall, these leaves (GBE) need more study and there is a recommendation to put the physical parameters parallel to the clinical study.
